# Supplementary material for: Study on coal dust diffusion law and new pneumatic spiral spray dedusting technology at transfer point of mine cross roadway
Source: PLoS One. 2022 Aug 22;17(8):e0272304. doi: 10.1371/journal.pone.0272304 (PMC9394847; doi:10.1371/journal.pone.0272304)
Supplement: Attachment — Submitted filename: Responese to reviewers.docx [file pone.0272304.s001.docx]

Dear Editor,

I thank the editor for allowing me to revise the article to make him better. With the advice of the two experts, I have revised the issues they raised in turn and changed the article to the formatting requirements of PLOS ONE as requested by the editor. If there are still any problems, please let me know by the editor and I will make further changes, and here is my response to the two reviewers.

Yours sincerely,

Tianzhang

Reviewer #1

1. The inconsistency of proper names in Figure 1 suggests a correction.

**Answer**: Corrected
2. Some references are not cited in the article and need to be added.

**Answer**: Modified
3. Some of the images have blurred text resulting in the information in the images not being read by the reader and affecting the overall nature of the article. For example, Figure 8, Figure 10.

**Answer**: The picture has been modified clearly
4. The text has a large number of images applied to the wind flow field at the point of reproduction, it is recommended that the images be combined or selectively deleted.

**Answer**: Figure 9 and Figure 10 have been combined into a new Figure 9, entitled Wind flow distribution characteristics and wind flow line trajectory, which allows the reader to understand the wind flow field more intuitively.
5. Boundary conditions are very important for numerical simulations anddifferent boundary conditions can affect the final results of the simulation, therefore boundary conditions should be specified. It is recommended to add dust source conditions and droplet atomisation conditions.

**Answer**: The dust source condition and the droplet atomisation condition have been added to the boundary conditions in Table 1.
6. The authors' figure 12 reflects the spiral spray velocity field wind flow curve and is missing units. The dust hood is also a closed device and when spraying for a longer period of time there should be a build-up of droplets and wind flow at the bottom, whether the author's device has an opening should be clarified.

**Answer**: The unit has been added, on the new manuscript figure 11, and it has been added at the end of 3.3.2 that the unit has an open shape.
7. In the design of the treatment solution in paper 4.1, the authors demonstrate that the spray device is effective in capturing small particle size coal dust by measuring the droplet size with a spray laser particle size meter, the basis of which needs to be detailed.

**Answer**: The revision has been completed and the data measured by the instrument and plotted in Fig.3Structure diagram of pneumatic spiral nozzle proves that the spray atomisation is effective in capturing coal dust particles with droplets less than 30 μm in size accounting for more than 80% of the overall.
8. Authors should use superscripts for numbers in m³ in their conclusions.

**Answer**: The units have been revised.
9. The description of the experimental section is too brief and does notindicate a clear method of experimentation.

Answer: Added in Figure 6

Reviewer #2

1. Please compare the two groups of experiments in section 3.3.4 with charts.

**Answer**: Additional data changes have been made in Figure 6.
2. Why is the wind speed at the lower part of the tunnel greater than that at the upper part?

**Answer**: It has been added in the second sentence of the first paragraph of 4.1 that the wind speed in the lower part of the tunnel is greater than that in the upper part due to the influence of the support bars below the belt.
3. What are the characteristics of wind speed distribution in different zonal areas in horizontal and vertical directions?

**Answer**: The wind flow in the different zones is characterised by a greater wind speed above than below and a greater wind speed in the outlet direction than in the inlet direction.
4. Put forward reasonable suggestions according to the numerical simulation results.

**Answer**: The beginning of 4.3 has been revised to add a rationalisation proposal based on the results of the wind flow distribution and particle trajectories from the tunnel simulation, a dust cover device is proposed for full vortex coverage.
5. It is suggested that the findings of this study be discussed more in the summary.

**Answer**: Conclusions on dust cover mist droplets have been added to conclusion 3.
6. There are insufficient references, so more references need to be supplemented. The background and mechanism of seepage are not introduced clearly. In particular, The failure and damage characteristics of rock should be furtherdescribed. The author should introduce this mechanism.

**Answer**: The literature has been added and corresponds to it. As this article focuses on the dust transport pattern at the roadway transfer point and the application of the control device, for the damage of the rock is not the focus of this article, we will continue to follow up this direction in the next research and thank the experts for their comments.
