# Supplementary material for: Study on coal dust diffusion law and new pneumatic spiral spray dedusting technology at transfer point of mine cross roadway
Source: PLoS One. 2022 Aug 22;17(8):e0272304. doi: 10.1371/journal.pone.0272304 (PMC9394847; doi:10.1371/journal.pone.0272304)
Supplement: Attachment — Submitted filename: Responese to reviewers.docx [file pone.0272304.s002.docx]

Dear Editor,

I thank the editor for allowing me to revise the article to make him better.

Yours sincerely,

Tianzhang

Reviewer #4: The subject of this paper is about the control of dust pollution at the coal transfer point of the mine, which is studied by the finite element-dynamic mesh modeling method, and the wind flow field and dust particle trajectory at the transfer point site are simulated by the simulation COMSOL software, and the reliability of the simulation is verified through experiments and other perspectives. The wind flow field and particle trajectory of the simulated coal transfer site are studied to obtain the vortex distribution law, and the dust cover device is proposed to control the law. The idea is very novel, the arguments are more solid and the data are reliable. I think the research results can provide valuable reference for the research of dust pollution control at the coal transfer point of mine. There is no problem with the macroscopic nature of the overall article, there are some problems that need to be revised, and I recommend publishing this article if the following problems are solved.
1. The specific location of the coal transfer point should be shown in the roadway distribution system map of picture 1.

**Answer**:Modification completed
2. 3.3.1 The experimental part of the device is recommended to be put to the later reliability verification.

**Answer**:Modified and added to the current paragraph 3.4, combined with the original 3.3.4 into one paragraph
3. 3.3.2 The content lacks the specific description of the device dimensions.

**Answer**:Modified, in what is now paragraph 3.3.1
4. The content of 3.3.3 does not match with the described picture and needs to be revised.

**Answer**:Modified, in what is now paragraph 3.3.2
5. The font in Figure 11 is different from the font of other pictures, and it is suggested to be modified.

**Answer**:Modification completed
6. 4.3 The content analysis is less, and it is suggested to add specificity.

**Answer**:Modification completed
7. Conclusion 1 suggests specific description. And there is no serial number.

**Answer**:Modification completed
